# Two Half-Size ATP-Binding Cassette Transporters Are Implicated in Aluminum Tolerance in Soybean

**DOI:** 10.3390/ijms251910332

**Published:** 2024-09-26

**Authors:** Junjun Huang, Huanan Li, Yiwei Chen, Xiaoyu Li, Ziyu Jia, Kunxia Cheng, Luyu Wang, Huahua Wang

**Affiliations:** 1 College of Life Sciences, Henan Normal University, Xinxiang 453007, China; huangjj04@163.com (J.H.); hnli2022@163.com (H.L.); yiweichen2022@163.com (Y.C.); xyli2021@163.com (X.L.); ziyujia202404@163.com (Z.J.); kxcheng2023@163.com (K.C.); luywang2023@163.com (L.W.); 2 The Observation and Research Field Station of Taihang Mountain Forest Ecosystems of Henan Province, Xinxiang 453007, China

**Keywords:** aluminum tolerance, ABC transporter, cell wall, soybean

## Abstract

Aluminum (Al) toxicity severely restricts plant production in acidic soils. ATP-binding cassette (ABC) transporters participate in plant tolerance to various environmental stresses. However, ABC transporters implicated in soybean Al tolerance are still rare. Here, we functionally characterized two half-size ABC transporters (GmABCB48 and GmABCB52) in soybean. Expression analysis showed that *GmABCB48* and *GmABCB52* were induced only in the roots, especially in the root tips. Both GmABCB48 and GmABCB52 were localized at the plasma membrane. Overexpression of *GmABCB48* or *GmABCB52* in *Arabidopsis* reduced Al accumulation in roots and enhanced Al tolerance. However, expression of *GmABCB48* or *GmABCB52* in yeast cells did not affect Al uptake. Furthermore, transgenic lines expressing *GmABCB48* or *GmABCB52* had lower Al content in root cell walls than wild-type plants under Al stress. Further investigation showed that the Al content in cell wall fractions (pectin and hemicellulose 1) of transgenic lines was significantly lower than that of wild-type plants, which was coincident with the changes of pectin and hemicellulose 1 content under Al exposure. These results indicate that GmABCB48 and GmABCB52 confer Al tolerance by regulating the cell wall polysaccharides metabolism to reduce Al accumulation in roots.

## 1. Introduction

Aluminum (Al) is the most abundant metal element in the Earth’s crust and is widely recognized as the primary limiting factor of crop productivity in acidic soils [1]. Acidic soils account for approximately 50% of the world’s cultivated lands [2]. Al exists largely as non-toxic aluminosilicates or oxides, which are biologically inactive in neutral form [3]. In soils with a pH below 5.5, however, the insoluble Al compounds can be transformed into a higher active form of Al^3+^, which hinders the acquisition of water and nutrients as a result of inhibition of plant root elongation [4]. The initial and distinct symptom of Al toxicity is on root growth, leading to changes in root morphology, such as atrophy of root hairs, swelling of root tips, and decreased cell wall elasticity and plasticity [5,6]. Previous studies proposed that Al binding to the cell wall is a prerequisite for Al toxicity in plants [6,7]. Al accumulation in the cell wall destroys the structure of the cell wall, accompanied by reduced elasticity and plasticity, which explains the reason for inhibiting the elongation of root cells [6,7].

To resist Al toxicity, plants have mainly adopted two strategies for detoxifying Al, namely, external exclusion and internal tolerance mechanisms [2,8]. External exclusion is based on the exclusion of Al from the root symplast, such as releasing organic acid anions (citrate, malate, and oxalate) from roots to chelate Al and form non-phytotoxic Al, as well as modulating the rhizosphere pH to reduce the bioavailability of Al [9,10]. Moreover, lowering the deposition of Al in the root cell wall is also an important mechanism for reducing Al toxicity in plants [11,12]. It has been proven that Al^3+^ passing through the rhizosphere can bind to the root cell wall and have a toxic effect on plants [13]. Plants could develop Al tolerance by slashing the adsorption capacity of the root cell wall for Al [14,15]. Additionally, internal detoxification mainly includes sequestering or isolating Al to vacuoles and other areas insensitive to Al [16,17]. AtALS1 (aluminum sensitive 1) and OsALS1, two tonoplast-localized ATP-binding cassette (ABC) transporters, participate in the internal detoxification via the compartmentalization of Al into the vacuole in Arabidopsis [18] and rice [19], respectively. Therefore, identifying key genes implicated in the detoxification mechanisms is critical for breeding Al-tolerant varieties and improving crop yield in acidic soils.

The ABC transporter family is widely present in living organisms and predominantly in land plants [20]. ABC transporters generally contain two domains: the hydrophobic transmembrane domain (TMD), containing four to six transmembrane helices, and the nucleotide-binding domain (NBD), containing several highly conserved characteristic motifs, such as Walker A motif [GXXGXGKS/T], Walker B motif [(RK)X_3_GX_3_L(hydrophobic)_3_], ABC signature motif, the Q-loop, and the H-loop [21]. TMDs serve as recognition agents and channels for substrate transport lipid bilayers, whereas NBDs provide energy for substrate transport or non-transport processes through ATP binding and ATP hydrolysis [21]. Most ABC transporters have both TMDs and NBDs, forming full-size proteins (two TMDs and two NBDs) or half-size proteins (one TMD and one NBD) [22]. On the basis of the phylogenetic analysis of ABC transporters, the similarity of conserved sequences, and the organization of their domains, plant ABC transporters are divided into eight subfamilies: ABCA—ABCG and ABCI [23]. The ABC transporter family is connected with many physiological processes in plants, including transporting plant hormones or metals, secreting secondary metabolites, and resisting various stresses [24]. ABC transporters are also implicated in the detoxification of Al in plants. In rice, ABC transporters OsSTAR1 (sensitive to Al rhizotoxicity 1) and OsSTAR2 are involved in Al tolerance, probably by affecting cell wall modification [25]. FeSTAR1/FeSTAR2 in buckwheat [26] and AtSTAR1/AtALS3 in Arabidopsis [27,28] are corresponding homologs of OsSTAR1/OsSTART2, respectively, which show a similar Al tolerance function. Furthermore, the half-size ABC transporters AtALS1 and OsALS1 confer the detoxification of Al in Arabidopsis and rice via vacuolar Al sequestration, respectively [18,19]. Similarly, buckwheat ABC transporters FeALS1.1 and FeALS1.2 are also implicated in Al tolerance by isolating Al into vacuoles [29]. However, there are few reports on the ABC transporters associated with Al tolerance in soybeans, especially the ABCB transporter subfamily.

Soybean is one of the most important oil and protein crops in the world. Due to its efficient nitrogen fixation ability, soybeans are considered a pioneer crop for improving the fertility of acidic soils. Therefore, exploring Al tolerance genes in soybeans is of great significance for sustainable agricultural productivity. We previously identified 255 ABC transporter members from the soybean genome and found that GmABCB48 and GmABCB52 are located on the same evolutionary branch with OsALS1 and AtALS1 [30]. Therefore, we hypothesized that GmABCB48 and GmABCB52 are implicated in Al detoxification. In this study, we functionally characterized GmABCB48 and GmABCB52 and found that they function in Al tolerance by regulating cell wall modification to reduce Al deposition in root cell walls.

## 2. Results

### 2.1. Sequence Analysis of GmABCB48 and GmABCB52 in Soybean

**Figure 1:**
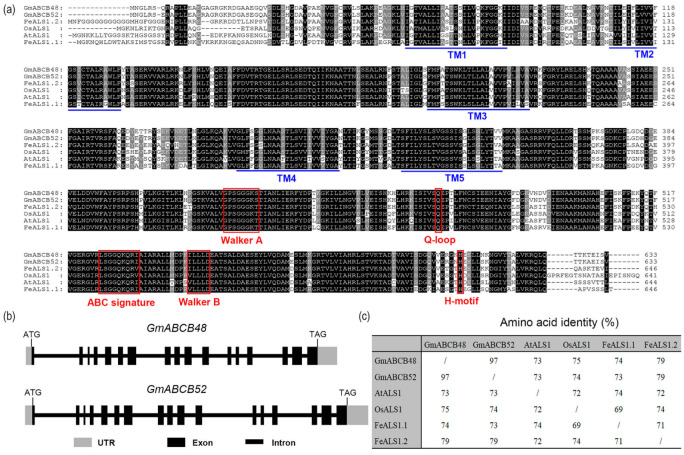
Multiple alignment and sequence analysis of GmABCB48 and GmABCB52. (**a**) Sequence alignment of GmABCB48, GmABCB52 and homologous proteins from other species, including *Arabidopsis thaliana* (AtALS1, At5g39040), *Oryza sativa* (OsALS1, Os03g0755100) and *Fagopyrum esculentum* (FeALS1.1, LC269045; FeALS1.2, LC269046). Blue horizontal lines indicate transmembrane domains). TM, transmembrane helix. Red boxes show the conserved motifs of the nucleotide-binding domain (NBD) in ABC transporters. (**b**) Gene structure of *GmABCB48* and *GmABCB52*. (**c**) Similarity of GmABCB48, GmABCB52, AtALS1, OsALS1, FeALS1.1 and FeALS1.2. All sequences were aligned by ClustalW. The protein domains were predicted by SMART.

The coding regions of both *GmABCB48* (Glyma.05G019400.1) and *GmABCB52* (Glyma.17G080400.1) consist of 1902 bps, which encodes a peptide of 633 amino acids (Figure 1a). Both *GmABCB48* and *GmABCB52* contain 17 exons and 16 introns (Figure 1b). At the amino acid level, GmABCB48 showed 97% identity with GmABCB52. Both GmABCB48 and GmABCB52 also showed 73–79% identity with OsALS1, AtALS1, FeALS1.1, and FeALS1.2 (Figure 1c). *GmABCB48* and *GmABCB52* encode a half-size ABC transporter, which contains typical ABC transporter motifs such as Walker A and B, a Q-loop, an H-motif, and an ABC signature (Figure 1a). Similar to AtALS1, OsALS1, FeALS1.1, and FeALS1.2, the TMDs of both GmABCB48 and GmABCB52 were also predicted to have five transmembrane helices (Figure 1a).

### 2.2. Expression Pattern of GmABCB48 and GmABCB52

**Figure 2:**
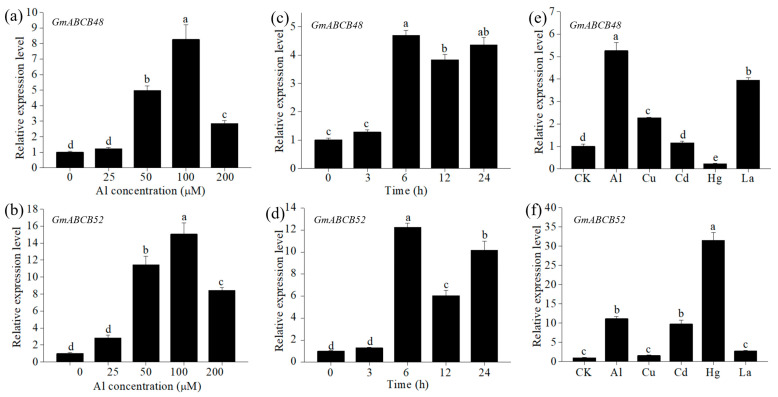
Expression pattern of *GmABCB48* and *GmABCB52*. (**a**,**b**) Dose-response expression of *GmABCB48* and *GmABCB52* to Al exposure for 6 h in soybean roots. (**c**,**d**) Time-course expression of *GmABCB48* and *GmABCB52* in roots of soybean treated with 100 μM of Al. (**e**,**f**) Metal-response expression of *GmABCB48* and *GmABCB52* in roots of soybean exposed to 50 μM of Al, 0.5 of μM Cu, 25 μM of Cd, 10 μM of Hg or 10 μM of La for 6 h. The data represent means ± SD. Different letters above the bars indicate significant differences (*p* < 0.05, LSD test).

**Figure 3:**
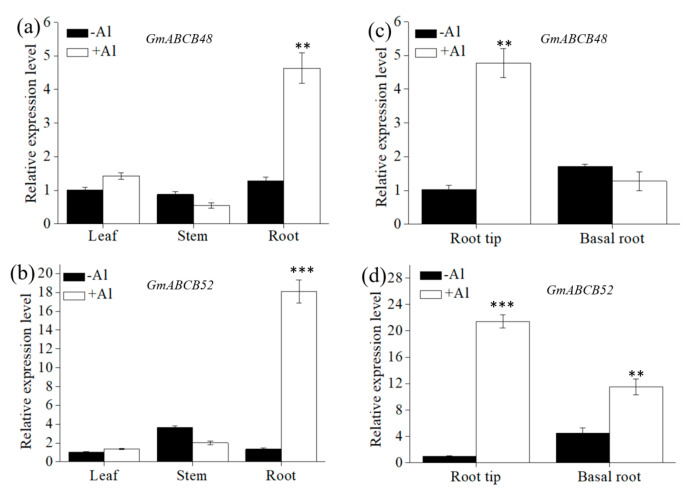
Tissue specificity of *GmABCB48* and *GmABCB52* expression. (**a**,**b**) Tissue-specific expression of *GmABCB48* and *GmABCB52* in leaves, stems, and roots of soybean in the presence of 0 or 100 μM of Al for 6 h. (**c**,**d**) Root spatial expression of *GmABCB48* and *GmABCB52* in root tips and basal roots exposed to 0 or 100 μM of Al for 6 h. Data represent means ± SD. Asterisks above the bars indicate a significant difference compared with the control as determined by Student’s *t*-test (** *p* < 0.01, *** *p* < 0.001).

In a dose-response experiment, the expression levels of both *GmABCB48* and *GmABCB52* gradually increased with increasing Al concentration and reached a maximum at 100 μM of Al, approximately 8 and 15 times that of the control, respectively (Figure 2a,b). A time-course experiment revealed that the induction of both *GmABCB48* and *GmABCB52* expression occurred at 6 h after Al exposure (Figure 2c,d). To investigate the specificity of *GmABCB48* and *GmABCB52* expression to Al stress, we compared the response of these genes to other metals. The expression of *GmABCB48* in roots could also be induced by Cu and La, approximately 2.3 and 3.9 times that of the control, respectively, while *GmABCB52* expression could be upregulated by Cd and Hg, approximately 9.8 and 31.5 times that of the control, respectively (Figure 2e,f). Tissue-specific expression demonstrated that both *GmABCB48* and *GmABCB52* expression could be detected in leaves, stems, and roots under normal conditions; however, the expression was only upregulated in the roots under Al exposure (Figure 3a,b). Spatial expression showed that both *GmABCB48* and *GmABCB52* expression was mainly induced in the root tips under Al exposure (Figure 3c,d).

### 2.3. Subcellular Localization of GmABCB48 and GmABCB52

**Figure 4:**
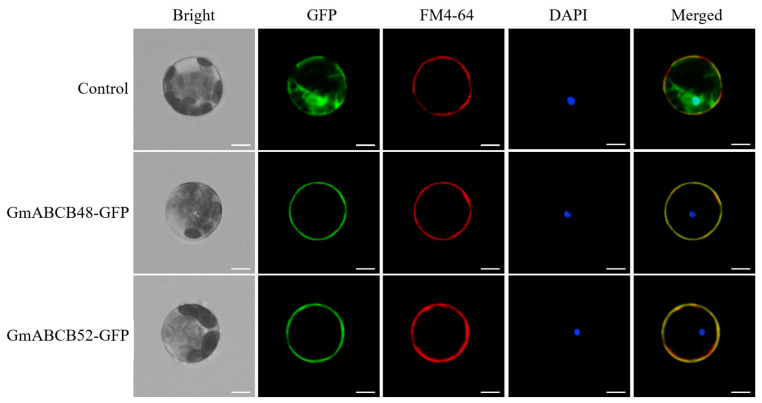
Subcellular localization of GmABCB48 and GmABCB52 in Arabidopsis leaf protoplasts. The first, second, and third rows show representative protoplasts transformed with the empty vector as a control, *GmABCB48-GFP* and *GmABCB52-GFP*, respectively. The first column shows a bright-field image of each protoplast; the second column shows GFP fluorescence patterns; the third column shows FM4-64 plasma membrane stain fluorescence patterns; the fourth column shows DAPI nucleus stain fluorescence patterns; the fifth column shows an overlay of the fluorescent images. Scale bar = 10 μm.

To detect the subcellular localization, a GmABCB48-GFP or GmABCB52-GFP fusion protein was transiently expressed in *Arabidopsis* leaf protoplasts. The GFP signal in the control protoplasts was observed at the plasma membrane, cytoplasm, and nucleus. In contrast, the GFP signal of GmABCB48-GFP or GmABCB52-GFP fusion was localized at the plasma membrane (Figure 4). To further verify the localization of the GmABCB48 and GmABCB52, the fluorescent dye FM4-64, which specifically labels the plasma membrane, was used. As shown in Figure 4, the GFP signal of GmABCB48-GFP or GmABCB52-GFP co-localized with FM4-64 fluorescence. These observations indicate that GmABCB48 and GmABCB52 are localized at the plasma membrane.

### 2.4. GmABCB48 and GmABCB52 Confer Al Tolerance

**Figure 5:**
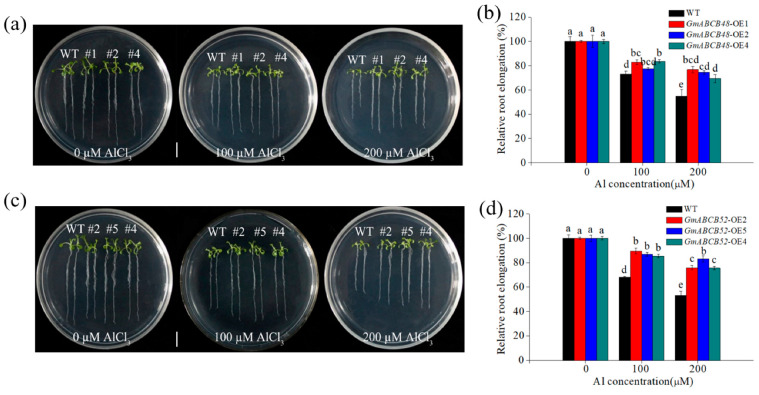
Transgenic Arabidopsis plants expressing *GmABCB48* or *GmABCB52* enhanced Al tolerance. Phenotype (**a**,**c**) and relative root elongation (**b**,**d**) of transgenic Arabidopsis and WT plants exposed to 100 and 200 μM of AlCl_3_ for 7 d on an agar plate. Scale bar = 1 cm. Relative root elongation was defined as the percentage of the root elongation of the treatment compared with the control. Data are means ± SD. Different letters above the bars indicate significant differences (*p* < 0.05, LSD test). WT, wild type.

To evaluate the roles of *GmABCB48* and *GmABCB52* in Al tolerance, we constructed transgenic Arabidopsis lines expressing *GmABCB48* or *GmABCB52*. Three homozygous T3 lines of *GmABCB48* (OE1, OE2 and OE4) and *GmABCB52* (OE2, OE4 and OE5) were verified by RT-PCR. RT-PCR analysis showed that *GmABCB48* and *GmABCB52* were transcribed in three transgenic lines, whereas they were absent in wild-type (WT) plants (Figure S1). Root elongation inhibition is a characteristic indicator of Al toxicity in plants. As shown in Figure 5a–d, root growth was similar between WT and transgenic plants overexpressing *GmABCB48* or *GmABCB52* in the absence of Al. However, under Al stress, the root elongation in WT plants was inhibited more than that of transgenic lines (Figure 5a–d). For example, under 200 μM of Al exposure, the relative root elongation of WT, *GmABCB48*-OE1, *GmABCB48*-OE2, and *GmABCB48*-OE4 was 54.9, 76.7, 74.6, and 69.5%, respectively, while that of WT, *GmABCB52*-OE2, *GmABCB52*-OE4, and *GmABCB52*-OE5 was 53.1, 75.9, 75.6, and 83.0%, respectively. These results indicate that overexpression of *GmABCB48* or *GmABCB52* improves the Al resistance of transgenic plants.

### 2.5. GmABCB48 and GmABCB52 Reduce Al Accumulation in Root Tips

**Figure 6:**
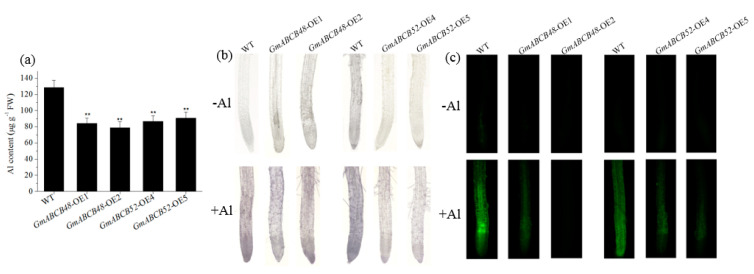
Al content in roots of transgenic Arabidopsis and WT plants. (**a**) Al content in roots of WT and transgenic lines after Al treatment. (**b**) Hematoxylin staining of Al in roots of WT and transgenic lines. (**c**) Morin staining of Al in roots of WT and transgenic lines. Four-week-old Arabidopsis seedlings were exposed to 0 or 50 μM of an AlCl_3_ solution containing 0.5 mM of CaCl_2_ (pH 4.5) for 6 h. WT, wild type. Asterisks above the bars indicate a significant difference compared with WT plants as determined by Student’s *t*-test (** *p* < 0.01).

The accumulation of Al in roots is closely related to Al toxicity. To investigate whether GmABCB48 and GmABCB52 regulate Al accumulation, the Al content in root tips was determined. Compared with WT plants, the Al content in transgenic Arabidopsis lines expressing *GmABCB48* or *GmABCB52* was decreased by 29.7–38.7% under Al stress (Figure 6a). Hematoxylin staining showed that the transgenic lines expressing *GmABCB48* or *GmABCB52* accumulated less Al in root tips than the WT plants under Al exposure (Figure 6b). Furthermore, the Al content in the roots was further labeled using Al fluorescence dye morin. As shown in Figure 6c, the fluorescence signals in the roots of transgenic lines were weaker than that in WT plants. These results indicate that the enhanced Al tolerance of transgenic plants is probably due to a reduction in root tip Al accumulation.

### 2.6. Al Transport Activity of GmABCB48 and GmABCB52

The transport activity of GmABCB48 and GmABCB52 was detected using a yeast expression system. RT-PCR analysis showed that *GmABCB48* and *GmABCB52* were transcribed in transgenic yeast lines, whereas they were absent in the control cells (Figure S2). Under normal conditions, the growth was similar between the control and yeast cells expressing *GmABCB48* or *GmABCB52* (Figure S3a). However, under Al stress, although the growth of both control and transgenic yeast cells was inhibited, there was also no notable difference between them (Figure S3b–d). Furthermore, the growth curves in transgenic yeast cells, with or without Al stress, also displayed a similar growth to that of control cells (Figure S3e), suggesting that expression of *GmABCB48* or *GmABCB52* in yeast did not affect its Al tolerance. The uptake of Al in yeast cells was further tested. As shown in Figure S3f, the Al content was similar between the control and transgenic yeast cells. These results imply that GmABCB48- and GmABCB52-mediated Al tolerance may not be attributed to Al transport.

### 2.7. GmABCB48 and GmABCB52 Reduce Al Binding to Cell Wall

To examine the function of GmABCB48 and GmABCB52, Al content in the root cell wall was detected. Under Al exposure, transgenic Arabidopsis lines expressing *GmABCB48* or *GmABCB52* accumulated less Al than WT plants, approximately 30% and 33% lower than that of WT plants, respectively (Figure S4a,b), suggesting that transgenic plants could decrease Al accumulation in roots by reducing Al binding to the cell wall. Pectin and hemicellulose (HC) are the main target sites for Al binding in the cell wall. To dissect which component contributes to the enhanced Al binding to the cell wall, we further tested the Al content in different cell wall polysaccharides. Compared with WT plants, the Al content in pectin and HC1 of *GmABCB48-OE* and *GmABCB52-OE* significantly decreased under Al stress (Figure S4c–f). However, the Al content in HC2 was similar between WT and transgenic plants (Figure S4g,h).

**Figure 7:**
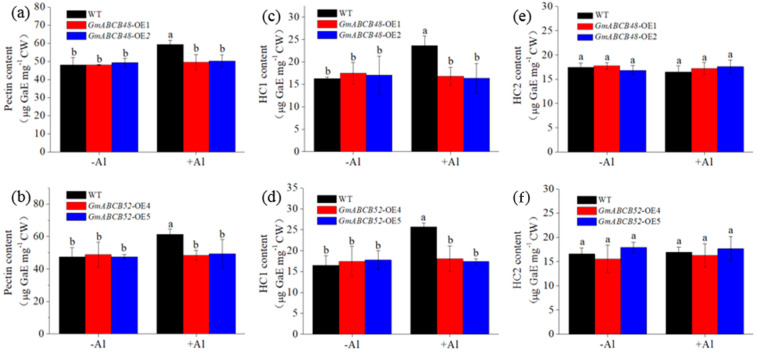
Effect of Al stress on the contents of cell wall pectin (**a**,**b**), HC1 (**c**,**d**), and HC2 (**e**,**f**) in roots of transgenic Arabidopsis and WT plants. Four-week-old Arabidopsis seedlings were exposed to 0 or 50 μM of an AlCl_3_ solution containing 0.5 mM of CaCl_2_ (pH 4.5) for 24 h. Data represent means ± SD. Different letters above the bars indicate significant differences (*p* < 0.05, LSD test). WT, wild type.

The contents of pectin, HC1, and HC2 were further determined. Under normal conditions, the content of each fraction in *GmABCB48-OE* and *GmABCB52-OE* was similar to WT plants (Figure 7a–f). However, under Al exposure, the contents of pectin and HC1 in transgenic lines were significantly lower than those in WT plants (Figure 7a–d), whereas HC2 content was similar between WT and transgenic plants (Figure 7e,f). These findings suggest that transgenic plants reduce Al deposition in cell walls by regulating the contents of pectin and HC1.

## 3. Discussion

Many reported studies have demonstrated that ABC transporters are connected with the tolerance of various stresses, such as drought, salinity, heavy metals, and Al toxicity [31,32,33]. ABC transporter genes associated with Al tolerance have been identified in several plant species, e.g., AtSTAR1 (AtABCI17)/AtALS3 (AtABCI16)/AtALS1 (AtABCB27) in Arabidopsis, OsSTAR1/OsSTAR2/OsALS1 (OsABCB25) in rice, and FeSTAR1/FeSTAR2/FeALS1.1/FeALS1.2 in buckwheat [18,19,25,26,27,28,29]. Among them, AtALS1, a half-size ABC transporter, is located in the tonoplast and is required for Al tolerance by isolating Al into vacuoles [18]. OsALS1 and FeALS1.1/FeALS1.2, the homologs of AtALS1, are also involved in Al detoxification via vacuolar Al sequestration in rice and buckwheat, respectively [19,29]. In spite of these, it is still elusive whether ABCB transporters could detoxify Al in soybeans. In this study, we identified two half-size ABC transporters, GmABCB48 and GmABCB52, which are the homologs of AtALS1/OsALS1 and implicated in Al tolerance. However, GmABCB48 and GmABCB52 show a distinct Al tolerance mechanism that is different from those of AtALS1/OsALS1.

The strategies for plants to resist Al toxicity include internal and external detoxification mechanisms [1,4]. Among them, the exudation of organic acid anions (citrate, malate, and oxalate), which is able to chelate Al externally, is the most well-studied strategy [10,34,35]. In soybeans, citrate transporter genes have been well characterized, e.g., *GmMATE13* and *GmMATE47* [36,37]. GmMATE-mediated citrate exudation is a crucial external mechanism of Al detoxification in soybeans, while this study displayed the existence of a GmABCB-mediated Al tolerance mechanism in soybeans. We demonstrated that GmABCB48 and GmABCB52 are the homologs of AtALS1 and OsALS1 (Figure 1). The functions of GmABCB48 and GmABCB52 in Al tolerance were verified in transgenic Arabidopsis (Figure 5 and Figure 6). Although GmABCB48 and GmABCB52 share 73–79% identity with their homologs from Arabidopsis, rice, and buckwheat (Figure 1), they have different Al tolerance mechanisms. It has been reported that root growth inhibition triggered by Al is related to the modification of root cell walls [38]. Here, we demonstrated that GmABCB48- and GmABCB52-mediated alleviation of Al toxicity could be attributed to changes in cell wall components. Our results showed that overexpression of *GmABCB48* or *GmABCB52* in Arabidopsis decreased root elongation inhibition and Al binding to cell walls compared to WT plants (Figure 5 and Figure S4).

The relative importance of symplastic versus apoplastic damage as a basis for Al toxicity remains a matter of debate. It has been proposed that the apoplast of the root apex is the primary site of Al toxicity [39]. In fact, there is ample experimental evidence that Al-induced root growth inhibition is associated with the disruption of cell wall functions [6]. More and more studies indicate that the cell wall is the main deposition site for Al in plants, and Al-induced root growth inhibition is associated with the change of cell wall properties, including cell wall viscosity and elasticity, and the content and chemical structure of the cell wall components [5,38,40,41,42]. Therefore, the exclusion of Al from root cell walls plays a pivotal role in plants resisting Al toxicity. The plant cell wall is mainly composed of pectin, HC, and cellulose. The negative charges on the pectin matrix, such as the free carboxyl groups of pectin, are the fundamental adsorption sites of Al^3+^ [43]. HC also plays an important role in the adsorption of Al^3+^, and HC1 is the largest accumulator of Al^3+^ in the cell wall [41]. Although the binding of Al in the cell wall has been proposed as a potential exclusion mechanism, it depends on the components that can bind Al. For example, the binding of Al to pectin contributes to Al toxicity in maize [44], while HC1 is correlated with Al accumulation in the cell wall and Al-induced root growth inhibition in Arabidopsis [41]. Additionally, in rice, a high Al content in root cell walls was attributed to abundant pectin and HC [42]. Here, we found that expressing *GmABCB48* or *GmABCB52* in Arabidopsis resulted in a significant decrease in cell wall Al accumulation in comparison with WT plants (Figure S4). More specifically, when different cell wall components were analyzed, GmABCB48 and GmABCB52 proteins consistently changed only Al accumulation in pectin and HC1 fractions, which was coincident with GmABCB48- and GmABCB52-mediated changes of pectin and HC1 contents (Figure 7 and Figure S4). Therefore, it implies that GmABCB48 and GmABCB52 regulate Al tolerance by specifically affecting the pectin and HC1 metabolism, which contributes to Al accumulation. Furthermore, we also found that the majority of Al in the cell wall was held by HC1, suggesting that HC1 contributes the most to Al accumulation in the cell wall. In rice, OsSTAR1/OsSTAR2 was found to form a complex to transport UDP-glucose, which is likely used to modify cell walls [25]. Similarly, the FeSTAR1–FeSTAR2 complex affects HC metabolism, probably via the transport of UDP-glucose, and thus regulates Al tolerance [26]. Therefore, whether GmABCB48 and GmABCB52 could transport UDP-glucose to modify cell walls needs further clarification in the future. Furthermore, as both of GmABCB48 and GmABCB52 are half-size ABC transporters, it will also be interesting to investigate whether GmABCB48 and GmABCB52 function as a homodimer or heterodimer.

In addition to distinct Al tolerance mechanisms, the expression patterns of *GmABCB48* and *GmABCB52* also differ from their homologs. *GmABCB48* and *GmABCB52* expression could be upregulated by Al in both roots and shoots (Figure 3), while *OsALS1* expression in roots was only upregulated by Al [19], and both *AtALS1* and *FeALS1.2* expression was unaffected by Al [18,29]. *GmABCB48* and *GmABCB52* were expressed in all tissues at a similar level (Figure 3), whereas *FeALS1.1* and *FeALS1.2* were expressed mainly in roots and leaves, respectively [29]. Furthermore, the expression of *AtALS1*, *OsALS1*, *FeALS1.1*, and *FeALS1.2* is specifically induced by Al [18,19,29]. In contrast, apart from Al, the expression of *GmABCB48* and *GmABCB52* could also be induced by other metals (Figure 2), suggesting that they may have unknown roles in response to other metals. These observations imply that the expression regulation mechanism appears to be different among these homologs. Furthermore, these homologs also differ in subcellular localization. AtALS1, OsALS1, FeALS1.1, and FeALS1.2 are localized to the tonoplast [18,19,29,45], but both GmABCB48 and GmABCB52 are localized to the plasma membrane (Figure 4). Therefore, the distinct Al tolerance mechanism between GmABCB48/GmABCB52 and their homologs may be attributed to the different expression patterns and subcellular localization.

In conclusion, GmABCB48 and GmABCB52 are plasma membrane-localized half-size ABC transporters. Both of them confer Al tolerance via regulating the pectin and HC1 contents in root cell walls.

## 4. Materials and Methods

### 4.1. Plant Growth and Treatment

Soybean (*Glycine max* L.) seeds of similar size were imbibed in water for 1 h and were then placed on a wet sponge to germinate. After germination, the seedlings were placed into plastic pots filled with 1/4-strength Hoagland nutrient solution for culture under controlled conditions (26 °C, 14/10 h photoperiod). Five-day-old seedlings were exposed to different treatment solutions containing CaCl_2_ (0.5 mM, pH 4.5). To examine the dose-response of gene expression, seedlings were treated with 0–200 μM of AlCl_3_ for 6 h. To explore the gene expression in response to different treatment times, plants were exposed to 100 μM of AlCl_3_ for 0–24 h. To examine the gene expression in response to different metals, seedlings were exposed to Al^3+^ (50 μM), Cu^2+^ (0.5 μM), Cd^2+^ (25 μM), Hg^2+^ (10 μM), and La^3+^ (10 μM) for 6 h. To investigate the gene expression in different tissues (leaf, stem, and root) and root segments (root tip and basal root), seedlings were treated with 0 or 100 μM of AlCl_3_ for 6 h.

### 4.2. Gene Expression Analysis

RNA was isolated using Trizol reagent (Takara, Japan), and cDNA was created from 1 μg of RNA with reverse transcriptase (Takara, Japan). A total of 1 μL of cDNA was added to the reaction mixture for PCR according to our previous study [46]. Primers used for quantitative RT-PCR are as follows: 5′-AATAACCAAACCCTACCACC-3′ and 5′-AAGGAACTTGTGCCTCAGT A-3′ for *GmABCB48*, 5′-GCAAATGTGGGATTCTGTAGGG’ and 5′-CAACTTTCCAGC CTCAGGTTTT-3′ for *GmABCB52*, and 5′-GGAAGGC TTTCTTGCATTGGTA-3′ and 5′-AGTGGCATCCT GGTACTC-3′ for *Tubulin3*. The gene expression level was normalized to the *Tubulin3* level. The relative expression values were calculated by formula 2^−ΔΔCT^.

### 4.3. Subcellular Localization

Subcellular localization was conducted as described by a previous study [47]. The CDS of *GmABCB48 and GmABCB52* was amplified with PCR primer pairs (5′-GTCGACATGAACGGCTTAAGAAGTCA-3′ and 5′-GGATCCGACCGAGATTTCGGTT TTTGTTG-3′ for *GmABCB48*, and 5′-GTCGACATGAACGGCTTAA GAAGTGAA-3′ and 5′-GGATCCAACCGAGATTTCGGCTTTTGTTG-3′ for *GmABCB52*). The PCR product was cloned into a 16318-hGFP vector to create 35S-GmABCBs-GFP. Then, the GmABCB48-GFP or GmABCB52-GFP fusion was transiently expressed in Arabidopsis leaf protoplasts. The fluorescence signals of GFP, plasma membrane dye FM4-64, and nucleus dye DAPI were observed using the confocal laser scanning microscope.

### 4.4. Overexpression of GmABCBs in Arabidopsis

The CDS of *GmABCB48* and *GmABCB52* were amplified with PCR primer pairs 5′-GTCGACGACCGAGATTTCGGTTTTT-3′ and 5′-GGATCCATGAACGGCTTAAGAA GTCA-3′, and 5′-GTCGACAACCGAGATTTCGGCTTTTG-3′ and 5′-GG ATCCATGAAC GGCTTAAGAAGTGA-3′, respectively. The PCR products were then cloned into the pCAMBIA1302-35S-EGFP vector. The constructs were transformed into Arabidopsis floral by EHA105 infiltration [48]. Homozygous T3 lines were selected with hygromycin, and the expression of *GmABCB48* and *GmABCB52* was confirmed by RT-PCR. Al tolerance evaluation of the transgenic lines was performed on an agar plate. Arabidopsis seeds were germinated on a 1/2 MS medium plate. Uniform seedlings with 0.5 cm root length were transferred to agar plates containing 0, 100, or 200 μM of AlCl_3_ (pH 4.5) for 7 d. Root length was measured before and after treatment.

### 4.5. Assay of Al Transport Activity

The CDS of *GmABCB48* and *GmABCB52* were amplified by PCR using primer pairs (5′-CTCGAGGACCGAGATTTCGGTTTTT-3′ and 5′-GGATCCATGAACG GCTTAAGAAGTCA-3′ for *GmABCB48*, and 5′-CTCGAGAACCGAGATTTCGG CTTTTG-3′ and GGATCCATGAACGGCTTAAGAAGTGA for *GmABCB52*) and then cloned into the pYES2 vector. The constructs GmABCB48-pYES2 and GmABCB52-pYES2 were transformed into BY4741 yeast cells. For Al tolerance evaluation, different concentrations of cell suspension were spotted on agar plates with 0, 100, 200, and 300 μM of AlCl_3_ and incubated for 3 d. To test the Al uptake, yeast cells (OD_600_ = 2.0) were treated with 50 μM of Al for 6 h.

### 4.6. Histochemical Staining

Four-week-old Arabidopsis plants were exposed to 50 μM of AlCl_3_ solution for 6 h. Hematoxylin staining was carried out according to our previous study [49]. Roots were rinsed with deionized water and then stained with 0.2% hematoxylin for 20 min. After washing off the excess dye on the surface, the roots were observed using a stereomicroscope (Nikon, Tokyo, Japan). Morin staining was performed as described by a previous study [50]. Roots were incubated with 0.01% morin for 20 min. The fluorescence signal was then observed using an epifluorescence microscope (Nikon, Tokyo, Japan).

### 4.7. Isolation of Cell WALL Components

Four-week-old Arabidopsis plants were exposed to a 50 μM AlCl_3_ hydroponics solution for 24 h. The extraction of cell wall components was conducted according to a previous work [51]. Roots were ground with liquid nitrogen and extracted with 75% ethanol on ice. After centrifugation (12,000× *g*, 4 °C) for 10 min, acetone, methanol-trichloromethane (1:1), and methanol was added to the precipitate in turn, and the precipitate obtained after centrifugation was the cell wall. The sample was dried and mixed with ammonium oxalate solution (0.5%). After boiling and centrifugation, the supernatant obtained was the pectin fraction. The above precipitate was incubated with 4 or 24% KOH containing 0.1% sodium borohydride for 12 h, and the supernatant was HC1 or HC2 fraction, respectively.

### 4.8. Analysis of Cell Wall Component Contents

The contents of cell wall fractions were conducted as described by a previous study [52]. Briefly, the pectin component (200 μL) was mixed with 3 mL of borate and 100 μL of 0.1% carbazole. The mixture was incubated for 1 h and then quickly cooled. Finally, the absorbance value was detected at 530 nm. Uronic acid was used to represent the content of pectin, and the determination of HC1 and HC2 was the same as that of pectin.

### 4.9. Measurement of Al Content

Al content was tested as described by the method of a previous study [11]. After digestion with HNO_3_ and HF, the Al content in cell wall components (pectin, HC1, and HC2) was analyzed by ICP-MS.

### 4.10. Statistical Analysis

All data were analyzed using ANOVA procedures using DPS software (Version 7.0), and LSD or Student’s *t*-test was used to test the differences among treatments at 0.05 level.

## Data Availability

The original datasets are presented in the article and Supplementary Materials. Further inquiries can be directed to the corresponding author.

## Supplementary Materials

The following supporting information can be downloaded at https://www.mdpi.com/article/10.3390/ijms251910332/s1.
