# Supplementary material for: Two Half-Size ATP-Binding Cassette Transporters Are Implicated in Aluminum Tolerance in Soybean
Source: Int J Mol Sci. 2024 Sep 26;25(19):10332. doi: 10.3390/ijms251910332 (PMC11476899; doi:10.3390/ijms251910332)
Supplement: Supplementary file 1 [file ijms-25-10332-s001.zip › ijms-3171400-supplementary.pdf]

**Supplementary Materials**

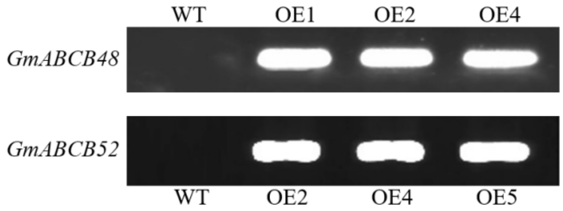


Figure S1. Expression analysis of *GmABCB48* or *GmABCB52* in three representative homozygous T3 lines expressing *GmABCB48* (OE1, OE2 and OE4) or *GmABCB52* (OE2, OE4 and OE5) and WT plants. RT-PCR was performed to detect the mRNA levels.

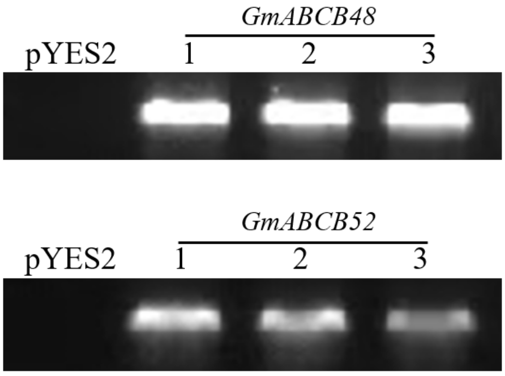


Figure S2. Expression analysis of *GmABCB48* or *GmABCB52* in yeast cells expressing *GmABCB48* or *GmABCB52* and control cells carrying empty vector. RT-PCR was performed to detect the mRNA levels.

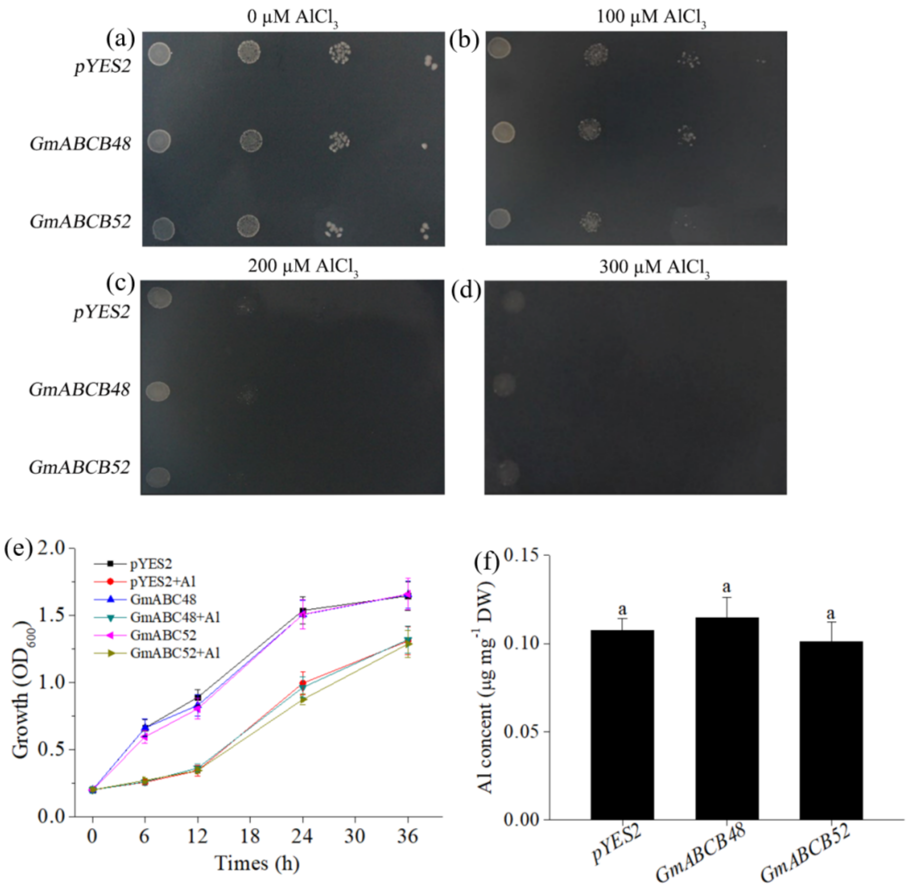


**Figure S3.** Al transport of GmABCB48 and GmABCB52 in yeast cells. **(a–d)** Al sensitivity of yeast cells carrying the pYES2 control vector, or GmABCB48- or GmABCB52-containing vectors on agar plates. Cell suspension ($OD_{600}$ = 2.0) and three serial 1:10 dilutions were spotted on plates containing different $AlCl_3$ concentrations for 3 d. **(e)** Yeast cells were grown at 30 °C in LPM liquid media and subjected to 100 μM of $AlCl_3$ at an $OD_{600}$ of 0.2. Cell density was monitored with the absorbance at 600 nm at 6, 12, 24, and 36 h after the Al treatment. **(f)** Al uptake in yeast cells carrying the pYES2 control vector, or GmABCB48- or GmABCB52-containing vectors. Yeast cells ($OD_{600}$ = 2.0) were exposed to 50 μM $AlCl_3$ for 6 h. Date represent means ± SD. Different letters above the bars indicate significant differences ($P < 0.05$, LSD test). WT, wild type.

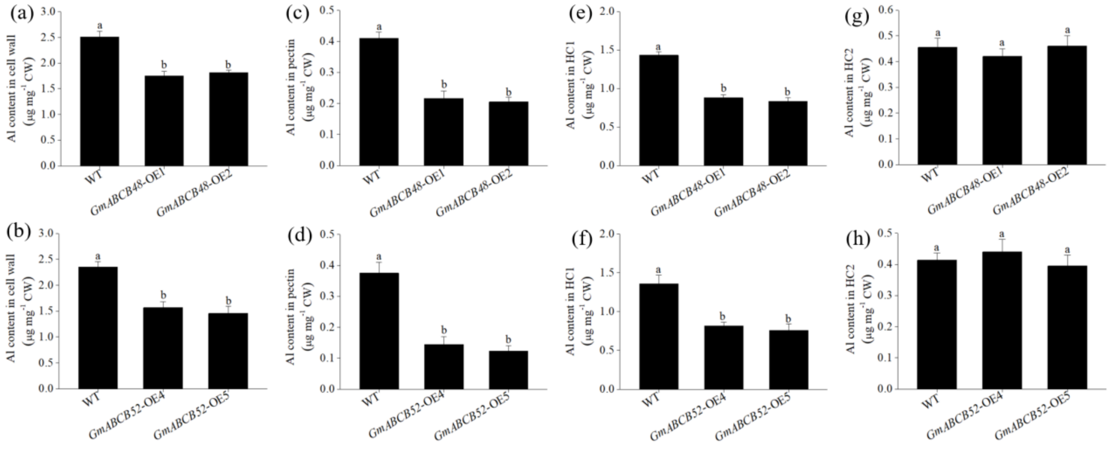


**Figure S4.** Al content in root cell wall **(a, b)**, pectin **(c, d)**, HC1 **(e, f)** and HC2 **(g, h)** of transgenic Arabidopsis and WT plants under Al stress. Four-week-old Arabidopsis seedlings were exposed to 50 μM $AlCl_3$ solution containing 0.5 mM $CaCl_2$ (pH 4.5) for 24 h. Date represent means ± SD. Different letters above the bars indicate significant differences (*P* < 0.05, LSD test). WT, wild type.
